# Measuring health literacy among older inpatients after elective surgery: evaluation of the European Health Literacy Survey Questionnaire-short form (HLS-EU-Q16)

**DOI:** 10.3389/fpsyg.2026.1935428

**Published:** 2026-08-28

**Authors:** Moritz Sebastian Schönfeld, Julia Rinke, Cynthia Olotu, Claudia Langebrake, Levente Kriston, Rainer Kiefmann, Corinna Bergelt

**Affiliations:** 1 Department of Medical Psychology, University Medical Center Hamburg-Eppendorf, Hamburg, Germany; 2 Hospital Pharmacy, University Medical Center Hamburg-Eppendorf, Hamburg, Germany; 3 Department of Anesthesiology, University Medical Center Hamburg-Eppendorf, Hamburg, Germany; 4 Department of Stem Cell Transplantation, Hospital Pharmacy, University Medical Center Hamburg-Eppendorf, Hamburg, Germany; 5 Department of Anesthesia, Rotkreuzklinikum München, Munich, Germany; 6 Department of Medical Psychology, University Medicine Greifswald, Greifswald, Germany

**Keywords:** confirmatory factor analysis, elective surgery, health literacy, HLS-EU-Q16, older adults, psychometry, reliability, validity

## Abstract

**Introduction:**

Health literacy (HL) has been recognized as a central construct in healthcare that contributes to individual health and serves as a predictor of various health outcomes. Recently, vulnerable groups such as older adults have increasingly become the focus of studies examining HL. In this context, various questionnaires have been developed and deployed, including the widely used European Health Literacy Survey Questionnaire (HLS-EU-Q47) and its short form (HLS-EU-Q16). However, it remains unclear whether results from population- and community-based studies can be applied to specific inpatient populations. Furthermore, research focusing on HL among older patients is limited. The aim of this study was therefore to examine distributions of HL across different characteristics and its associations with other health variables and to assess the psychometric properties of the HLS-EU-Q16 in a sample of older inpatients after elective surgery.

**Methods:**

We analyzed a cross-sectional sample of 143 hospital patients aged 65 years or older who underwent elective surgery at a university hospital in northern Germany. Levels of HL, along with various sociodemographic and health-related variables, were examined. Additionally, we conducted a confirmatory factor analysis (CFA) to examine the latent factor structure of the HLS-EU-Q16 in this context.

**Results:**

The average age of patients was 73.2 years (SD = 5.8), with 52.4% being male. Nearly half (47.6%) of the sample had difficulties in handling health information (i.e., limited HL). Overall, 13.8, 33.8, 40.0, and 12.3% of patients were classified as having inadequate, problematic, sufficient, and excellent HL, respectively. Patients reported the greatest difficulties with items related to mental health and media on the HLS-EU-Q16. No significant differences in HL levels were observed between age groups, genders, or other sociodemographic variables (*p* > 0.05). Regarding the CFA, we were unable to fully replicate the three-factor structure of the HLS-EU-Q16.

**Conclusion:**

Our findings align with previous research indicating high rates of limited HL among older adults. However, we were unable to confirm the three-factor model of the HLS-EU-Q16, which adds to the mixed findings across different participant groups and contexts of previous studies. Developing or adapting HL questionnaires for clinical purposes may improve their relevance and usability in future studies.

## 1 Introduction

Health literacy (HL) refers to an individual’s ability to find, access, and understand health information and to effectively use this information to improve one’s own health (Sørensen et al., 2012). Previous research has repeatedly emphasized the importance of HL, identifying it as a critical determinant of various health outcomes across different contexts and populations (Chesser et al., 2016; Berkman et al., 2011; Sørensen et al., 2015; Yu et al., 2025). Conversely, several predictors of limited HL (e.g., language skills, physical activity, and good over health) have also been described (Singh et al., 2022; The HLS19 Consortium of the WHO Action Network M-POHL, 2021). Despite numerous interventions and (inter)national strategies, recent studies from around the world continue to show that half or more of the adult population still have limited HL (Yu et al., 2025; Baccolini et al., 2021; Gonçalves-Fernández and Pino-Juste, 2025; de Jesus et al., 2024). This issue is particularly evident among individuals experiencing social, economic, or health burdens (Sørensen et al., 2015; Schaeffer et al., 2025). Older adults, in particular, are recognized as a highly vulnerable group (United Nations Department of Economic Social Affairs, 2020; World Health Organization, 2015).

Against the backdrop of global demographic aging, this population has received increasing scientific and clinical attention (World Health Organization, 2025). Older age is generally associated with higher health risks, multimorbidity, increased medication use, and more frequent hospitalizations (World Health Organization, 2015). As a result, many older adults require comprehensive care as their health needs become increasingly complex. In this context, HL plays a vital role in supporting effective health management (Cardoso et al., 2023). Despite advances in scientific research, most studies focus on community-dwelling older adults, and evidence in specific healthcare settings remains limited (Chesser et al., 2016). Existing research, however, suggests that low HL among older patients is associated with poorer postoperative outcomes, a higher risk of complications, and increased hospitalization rates (Nyman et al., 2018; Theiss et al., 2022; Lima et al., 2024). Therefore, measuring HL in hospitalized older adults may be crucial for identifying individual needs and facilitating faster recovery.

A variety of instruments, such as the European Health Literacy Survey Questionnaire (HLS-EU-Q47) (Sørensen et al., 2013), have been developed to assess HL. Because hospitalization is often accompanied by elevated stress and anxiety, brief self-report tools like the widely used HLS-EU-Q16 (short form of the HLS-EU-Q47), may offer a practical alternative to more demanding performance-based assessments. Different forms of the HLS-EU-Q are frequently used in research and practice, and across settings and demographic populations. Most research findings, however, come from studies in community-based settings (Gonçalves-Fernández and Pino-Juste, 2025; Bonaccorsi et al., 2025) since the HLS-EU-Q was initially developed to assess HL in larger populations (e.g., countries) (Sørensen et al., 2013). In addition, previous studies reported difficulties in replicating the factor structure of the HLS questionnaires in specific populations and settings, and psychometric differences in the development of country-specific versions of the questionnaire have been described (Gustafsdottir et al., 2020; Lamot and Kirbiš, 2024; Bergman et al., 2023; Finbråten et al., 2018; Coman et al., 2022). Moreover, as HL has been identified as a central determinant of health, clinical and policy decisions in this area are often directly impacted by available HL research from general population studies. In case of limited psychometric validity in these clinical situations, however, patients may, for example, show different responses to HL items or prioritize different aspects of HL due to the clinical situation. Resulting practical decisions may therefore not adequately fit patient needs and capabilities. Accordingly, these generic HL instruments should be validated within specific clinical populations and settings before clinical use. In addition, many population-based studies treat older adults as a homogeneous group, possibly overlooking meaningful differences between “young-olds,” “middle-olds,” and “old-olds” (Berkman et al., 2011; Bonaccorsi et al., 2025), potentially further limiting conclusions from available HL studies by overlooking age-specific variability. In summary, it remains uncertain whether these generic instruments are suitable for specific clinical environments or whether findings from population-based studies can be accurately replicated in such settings (Chesser et al., 2016).

Accordingly, the present study aimed to analyze HL among older patients in a clinical setting after elective surgery and to examine the psychometric properties of the HLS-EU-Q16. Specifically, we sought to explore response patterns and investigate the distribution of HL levels across various socioeconomic factors (e.g., education), personal characteristics (e.g., age), and health-related variables (e.g., medication adherence). To evaluate the psychometric properties of the questionnaire, we aimed to assess whether the factor structure of the HLS-EU-Q16 can be replicated within a clinical sample of older adults.

## 2 Materials and methods

### 2.1 Study design and data collection

Patients were recruited from the University Medical Center Hamburg-Eppendorf in Hamburg, Germany. Data collection occurred in two phases. First, we included control group data from the research project PHAROS, which aimed to examine the impact of a medication therapy management (MTM) intervention on medication appropriateness in older hospital patients after elective surgery. The PHAROS study followed a sequential intervention design in which data for the control group were collected first, followed by data collection for the intervention group. This approach was chosen because the intervention was expected to influence clinical practice, making sequential recruitment necessary to avoid confounding. Details of the PHAROS project are described elsewhere (Richter et al., 2020; Schönfeld et al., 2025). Subsequently, to reach an adequate sample size for this study, we recruited additional patients from surgical wards using a convenience sampling strategy. Only patients not enrolled in PHAROS were recruited during this phase. Inclusion criteria were identical for both samples: age 65 years or older, undergoing elective surgery within a few days prior to completing the questionnaires, and German-speaking. Exclusion criteria included severe cognitive impairment and psychiatric illness. We only included PHAROS control data to ensure comparability. Patients were recruited between June 2019 and July 2022. Due to the COVID-19 pandemic, recruitment was paused for approximately 1 year and interrupted multiple times. All participants completed a paper-and-pencil questionnaire that collected sociodemographic information and health outcomes, such as health literacy and medication adherence. Eligible patients were identified by medical staff and recruited by research personnel.

#### 2.1.1 Ethics approval

As this study contains data from two research projects, ethics approval was obtained by the Ethics Committee of the Hamburg Medical Association (no. PV5754, PHAROS study) as well as the local psychological ethics committee (no. LPEK-0064, convenience sample). All participants gave written informed consent and received additional verbal information about the rational, content, and use of their data.

### 2.2 Measures

#### 2.2.1 Health literacy

Self-reported HL was assessed using the HLS-EU-Q16, a short version of the HLS-EU-Q47 developed by the HLS-EU (Health Literacy Survey-Europe) consortium (Sørensen et al., 2012; Pelikan et al., 2014). Both instruments are based on the same conceptual model of HL. The HLS-EU-Q16 is a validated, multidimensional questionnaire and was established as Rasch homogenous (Röthlin et al., 2013). The underlying HL concept comprises four central skills (i.e., find, understand, appraise, and apply) across three health domains [health care (HC), disease prevention (DP), and health promotion (HP)] (Sørensen et al., 2012; Sørensen et al., 2013). The HLS-EU-Q16 is a self-report tool asking participants to rate 16-items on a four-point scale from “very difficult” to “very easy” or answering “do not know” (counted as missing values).

#### 2.2.2 Sociodemographic and secondary measures

The questionnaire completed by patients also included sociodemographic variables (e.g., age, gender, education, and income) and a question regarding the number of regularly taken medications (i.e., “How many medications do you take regularly?”). Age subgroups were defined as follows: 65–69 years, 70–79 years, and 80 years and older. Monthly net household income was categorized into two groups approximately reflecting the mean net household income in Germany in 2021: below average (<4,000 EUR) and above average (≥4,000 EUR). Educational level was classified into three categories following the International Standard Classification of Education as presented in previous studies (Tiller et al., 2015; Vogt et al., 2018). We additionally included validated questionnaires and screeners to assess patients´ medication adherence (MARS-D) (Mahler et al., 2010), health status (SF-12) (Ware et al., 1994), depression and anxiety symptoms (i.e., psychological distress based on PHQ-4) (Löwe et al., 2010), and cognitive functioning (WHODAS 2.0) (Ustün et al., 2010).

### 2.3 Analyses

#### 2.3.1 Health literacy

Following the recommendations of the HLS-EU-Q16 authors, index scores were calculated for all respondents who answered at least 80% of the items (Pelikan et al., 2014). Item responses were analyzed as follows: First, the mean score for each item was calculated. Second, for easier comparison, mean scores were standardized into a scale according to the authors recommendations as follows (Pelikan et al., 2019): G-HLS = (*Mean-*1)*(50/3), where *Mean* represents the average of all available item responses for each participant. G-HLS scores ranged from 0 (lowest HL) to 50 (highest HL). Four HL levels were defined: inadequate (0–25), problematic (>25–33), sufficient (>33–42), and excellent (>42–50). G-HLS scores were also calculated for each of the three HL domains (HC, DP, HP).

#### 2.3.2 Data analyses

Categorical descriptive data were analyzed using frequencies (n, %) and range (Min, Max). Continuous data were analyzed using means and standard deviations. Associations between single items, overall HL scores (G-HLS), and HL subdomains (HC, DP, HP) were examined using Spearman correlation coefficients where appropriate. For G-HLS, the 25th and 75th percentiles were calculated. Differences between groups regarding HL levels were tested using the Kruskal-Wallis test. Descriptive analyses were restricted to complete cases. Because recruitment was interrupted during COVID-19 lockdowns, we compared participants enrolled before and after the lockdowns regarding HL levels and key sociodemographic variables (age, gender, living situation, income, education).

All categorical HL items were treated as ordinal variables. Polychoric correlations and the diagonally weighted least squares (WLSMV) estimator were used in the confirmatory factor analysis (Li, 2016; Flora and Curran, 2004; Rogers, 2024). Missing values were handled using pairwise deletion because maximum likelihood estimation, although generally recommended, is neither available nor advised for CFA with ordinal data in R (Rogers, 2024; Lei and Shiverdecker, 2020). To evaluate construct validity of the polytomous item models (4-point Likert-type items), two models were specified in accordance with the conceptual framework of the HLS-EU-Q16: a unidimensional model (model 1, HL as a single latent factor) and a three-factor model (model 2, representing the theoretical HL competences HC, DP, HP). Correlated residuals were included when conceptually justified to account for shared variance not captured by the latent factors.

*A priori* power analysis indicated that a minimum sample size of 113 patients would be sufficient to conduct CFA for the three-factor model with 16 indicator variables, assuming.80 power at *p* < 0.05 (RMSEA<0.06, df = 101) (Moshagen and Bader, 2023). Following recommended practices for CFA, we aimed to recruit at least 130 participants for this study (Moshagen and Bader, 2023). Model fit was evaluated using the following indices and cutoff criteria: Comparative Fit Index (CFI > 0.95), Tucker–Lewis Index (TLI > 0.95), root mean square error of approximation (RMSEA<0.06), and standardized root mean square residual (SRMR<0.08) (Hu and Bentler, 1999).

All descriptive analyses were conducted in IBM SPSS (version 28) and CFA analyses were conducted using packages lavaan (Rosseel, 2012) and lavaanPlot (Alex, 2021) in RStudio (version 2022.12.0), (R Core Team, 2025).

## 3 Results

A total of 146 patients completed the questionnaire (40 from the PHAROS control group, recruited before the COVID-19 lockdown in Germany; 106 from the second recruitment phase, a convenience sample recruited after the lockdowns). Three cases were excluded due to more than 30% missing values, resulting in a final sample of 143 hospital patients. Patients were between 65 and 90 years of age (Mean = 73.2, SD = 5.8), and 52.4% were male. The most prevalent age group was 70–79 years, representing 52.4% of the sample. For additional information, see Table 1.

**Table 1:** Sociodemographic characteristics of the study sample.

	Frequencies	%
Gender		
Women	64	44.8
Men	75	52.4
Missing	4	2.8
Age subgroups		
65–69	46	32.2
70–79	75	52.4
≥80	20	14.0
Missing	2	1.4
Ward		
General surgery	109	76.2
Gynecology	8	5.6
Urology	15	10.5
Gastroenterology	1	0.7
Ear, nose and throat	8	5.6
Oral and maxillofacial	2	1.4
Number of drugs taken		
0–5	69	48.3
6–10	41	28.7
11–15	10	7.0
>15	2	1.4
Missing	21	14.7
Smoking		
No	121	84.6
Yes	19	13.3
Missing	3	2.1
Living situation		
Alone	58	40.6
With someone	77	53.8
Missing	8	5.6
School degree		
Low	41	28.7
Medium	69	48.3
High	31	21.7
Missing	2	1.4
Household income after taxes		
Below average (<4.000EUR)	66	51.7
Above average (≥4.000EUR)	46	32.2
Missing	23	16.1

Sensitivity analyses indicated that the recruitment phase (pre- vs. post-lockdown) did not statistically significantly influence mean HL scores (*p* > 0.05). Likewise, no statistically significant differences were observed between the two recruitment phases regarding gender, living situation, income, or educational level. However, patients recruited before the lockdown were statistically significantly older on average compared with those recruited afterward (Mean = 75.1 vs. 72.4). To address this age difference between recruitment phases, we conducted age-stratified sensitivity analyses to assess appropriateness of data pooling for primary analyses. Mann–Whitney U test revealed no statistically significant differences between recruitment phases regarding G-HLS (*z* = −1.10, *p* = 0.270), and Spearman correlations between G-HLS and age in either of the recruitment groups and the pooled data sample were small and not statistically significant (PHAROS sample: *ρ* = 0.15, *p* = 0.351; convenience sample: *ρ* = −0.13, *p* = 0.182; pooled sample: *ρ* = −0.02, *p* = 0.821). Finally, age effects did not statistically significantly differ between recruitment phases (*β* = −0.36, SE = 0.24, *t* = −1.52, *p* = 0.131). These findings indicate comparability regarding HL between both groups and suggest appropriateness of data pooling for primary analyses.

### 3.1 Descriptive analyses of health literacy

#### 3.1.1 Item responses

Table 2 presents the item responses to the HLS-EU-Q16. Non-response rates ranged from approximately 1% (Q3, Q4) to 25% (Q8), including “do not know” response ranging from 0% (Q2, Q3, Q4) to about 20% (Q8). Highest proportion of “do not know” responses were observed among items concerning mental health (Q8, Q13) and media (Q11, Q12, Q15) as well as advice from family and friends (Q14). Overall, items showed 6.5% missing responses on average. Items Q8, Q11, Q12, and Q15 showed the highest proportions of “difficult” responses (38.0 to 55.6%), with item means between 2.4 and 2.8. In contrast, items Q4, Q7, Q9, and Q10 exhibited the lowest proportions of “difficult” responses (3.7 to 9.2%), with item means between 3.0 and 3.5. At the item level, no floor or ceiling effects were observed on the 4-point response scale.

**Table 2:** Item responses for HLS-EU-Q16 items.

Domain	On a scale from very easy to very difficult, how easy would you say it is to…	Responses	Non-responses*		Mean and SD		“Fairly difficult” or “very difficult”		“Very easy”	“Fairly easy”	“Fairly difficult”	“Very difficult”
		*n*	*n* (“do not know”)	% (*n* = 143)	Mean	SD	*n*	%	%	%	%	%
HC	Q1. … find information about symptoms of illnesses that concern you?	131	12 (8)	8.4 (5.6)	2.8	0.70	30	22.9	12.2	64.9	17.6	5.3
HC	Q2. … find out where to get professional help when you are ill?	141	2 (0)	1.4 (0)	3.1	0.80	25	17.7	28.4	53.9	12.1	5.7
HC	Q3. … understand what a doctor says to you	142	1 (0)	0.7 (0)	3.0	0.70	25	17.6	23.2	59.2	14.8	2.8
HC	Q4. … understand your doctor’s or pharmacist’s instruction on how to take a prescribed medicine?	142	1 (0)	0.7 (0)	3.3	0.69	13	9.2	40.1	50.7	7.0	2.1
HC	Q5. … judge if you may need to get a second opinion from another doctor?	134	9 (7)	6.3 (4.9)	2.7	0.75	49	36.6	13.4	50.0	32.1	4.5
HC	Q6. … use information your doctor gives to you to make decisions about your illness?	140	3 (2)	2.1 (1.4)	2.9	0.73	38	27.1	16.4	56.4	23.6	3.6
HC	Q7. … act on advice from your doctor or pharmacist?	140	3 (2)	2.1 (1.4)	3.3	0.66	13	9.3	37.1	53.6	7.9	1.4
DP	Q8. … find information on how to handle mental health problems?	107	36 (28)	25.2 (19.6)	2.7	0.84	48	44.9	16.8	38.3	38.3	6.5
DP	Q9. … understand information about unhealthy habits such as smoking, low physical activity or drinking too much alcohol?	134	9 (5)	6.3 (3.5)	3.5	0.57	5	3.7	58.2	38.1	3.7	0.0
DP	Q10. … understand information about recommended health screenings or examinations?	141	2 (1)	1.4 (0.7)	3.0	0.62	8	5.7	66.7	27.7	5.0	0.7
DP	Q11. … judge if the information on health risks in the mass media is reliable?	133	10 (9)	7.0 (6.3)	2.4	0.77	74	55.6	6.0	38.3	44.4	11.3
DP	Q12. … decide how you can protect yourself from illness using information from the mass media?	132	11 (9)	7.7 (6.3)	2.6	0.80	66	50.0	13.6	36.4	43.9	6.1
HP	Q13. … find information about activities that are good for your mental health and well-being?	129	14 (11)	9.8 (7.7)	3.0	0.69	27	20.9	21.7	57.4	19.4	1.6
HP	Q14. … understand advice concerning your health from family or friends?	128	15 (13)	10.5 (9.1)	3.0	0.86	31	24.2	26.6	49.2	16.4	7.8
HP	Q15. … understand information in the mass media on how to improve your health?	129	14 (12)	9.8 (8.4)	2.8	0.76	49	38.0	16.3	45.7	34.9	3.1
HP	Q16. … judge which everyday habits affect your health?	137	6 (4)	4.2 (2.8)	3.3	0.68	17	12.4	38.0	49.6	11.7	0.7

*Non-responses include “do not know” answers and refusals to answer and are calculated based on the overall sample of *n*=143; HC, Health Care; DP, Disease Prevention; HP, Health Promotion.

#### 3.1.2 Health literacy levels

Overall, 47.6% (based on the G-HLS) of older patients in this study had limited HL levels (Table 3). The mean and median of the G-HLS were 33.2 (SD = 7.2) and 33.3 (P25: 29.2, P75: 38.5), respectively. According to the G-HLS, 13.8% of patients were classified as having inadequate HL, 33.8% as problematic, 40.0% as sufficient, and 12.3% as having excellent HL (Table 3).

**Table 3:** Overall and subgroup HL-level scores in 143 elderly inpatients after elective surgery.

Age group	65–69 years		70–79 years		≥80 years		Total				
	*n*	%	*n*	%	*n*	%	*n*	%	Mean (SD)	[CI]	Median
HL index score (G-HLS)									33.2 (7.2)	32.0–34.5	33.3
Inadequate health literacy	7	15.9	9	12.9	2	12.5	18	13.8	21.4 (4.2)	19.3–23.5	23.8
Problematic health literacy	15	34.1	23	32.9	6	37.5	44	33.8	29.5 (2.0)	28.9–30.1	31.0
Sufficient health literacy	19	43.2	26	37.1	7	43.8	52	40.0	36.8 (2.8)	36.0–37.6	37.1
Excellent health literacy	3	6.8	12	17.1	1	6.3	16	12.3	44.3 (1.8)	43.4–45.2	44.4

Sample size varies due to missing values.

#### 3.1.3 Health literacy domains and skills

Mean values across the three HL domains were comparable: HC (*M* = 33.7, SD = 8.9), DP (*M* = 32.7, SD = 8.3), and HP (*M* = 33.0, SD = 8.9). No statistically significant differences were observed between age subgroups (Supplementary Table 1). Likewise, analyses of the four HL skill areas showed no statistically significant differences across the three age subgroups (Supplementary Table 2). Across all age subgroups, participants reported the greatest difficulty with appraising health information (*M* = 29.7, SD = 8.9), while understanding health information posed the least difficulty (*M* = 36.6, SD = 7.6).

### 3.2 Variables associated with health literacy

We calculated Spearman correlation coefficients, which revealed statistically significant weak to moderate associations among most of the 16 individual items, as well as moderate to strong associations between the G-HLS and the three HL domains (HC, DP, HP) (Supplementary Tables 3, 4).

#### 3.2.1 Sociodemographic variables

Mean G-HLS scores ranged from 31.9 in the over-80 subgroup to 33.9 in the 70–79-year-old group. Mean G-HLS scores were also similar between genders (male: 32.8; female: 33.3). Overall, G-HLS scores showed no statistically significant differences across gender, age subgroups, educational levels, or monthly net household income (Supplementary Table 5). Likewise, no statistically significant differences were observed in these sociodemographic variables with respect to the three HL domains or the four HL skill areas.

#### 3.2.2 Health outcomes

Regarding health outcomes, 27 patients (19.7%) demonstrated non-adherent medication behavior as assessed by the MARS-D. Additionally, 22 patients (16.7%) reported moderate to severe symptoms of anxiety and depression according to the PHQ-4. Self-reported physical health, measured using the SF-12 (PCS), was relatively low (*M* = 35.9, SD = 10.5), whereas self-reported mental health, also assessed via the SF-12 (MCS), was comparatively higher (*M* = 48.8, SD = 11.9). Spearman correlations indicated statistically significant but weak associations between HL and other health outcomes, including anxiety and depression symptoms (PHQ-4), self-reported physical and mental health (SF-12 PCS and MCS), and cognitive functioning (WHODAS 2.0). Medication adherent behavior (MARS-D) showed a weak but significantly positive correlation with the HC dimension of HL (*p* < 0.05). Notably, G-HLS scores differed statistically significantly across levels of psychological distress and patients with higher distress exhibited lower HL scores (*p* = 0.02). For further details, see Supplementary Tables 5–7.

### 3.3 Confirmatory factor analysis

Confirmatory factor analyses were conducted to examine the factor structure of the HLS-EU-Q16. Both unidimensional and three-factor models were tested using polytomous item responses. Based on conceptual overlap, correlated residuals were included in secondary models for Q11, Q12, and Q15, reflecting shared variance among media-related items. Overall, none of the tested models demonstrated acceptable fit according to the predefined cut-off criteria (Table 4).

**Table 4:** Polytomous item models.

Dimensional structure	Model	χ^2^/df	*p*	CFI	TLI	SRMR	RMSEA	RMSEA CI
Unidimensional	Model 1.1	4.91	<0.001	0.81	0.78	0.16	0.17	0.15–0.18
	Model 1.2^†^	3.83	<0.001	0.87	0.84	0.15	0.14	0.13–0.16
Three-dimensional	Model 2.1	2.97	<0.001	0.91	0.89	0.13	0.12	0.10–0.13
	Model 2.2^†^	2.28	<0.001	0.94	0.93	0.11	0.10	0.08–0.11
Bi-factor	Model 3^†*^	1.46	0.004	0.98	0.97	0.05	0.06	0.03–0.08

^†^Correlated residuals between Q11-Q12, Q11-Q15, Q12-Q15 (i.e., media-related items) were included. *Exploratory model.

#### 3.3.1 Polytomous item models

**Figure 1:**
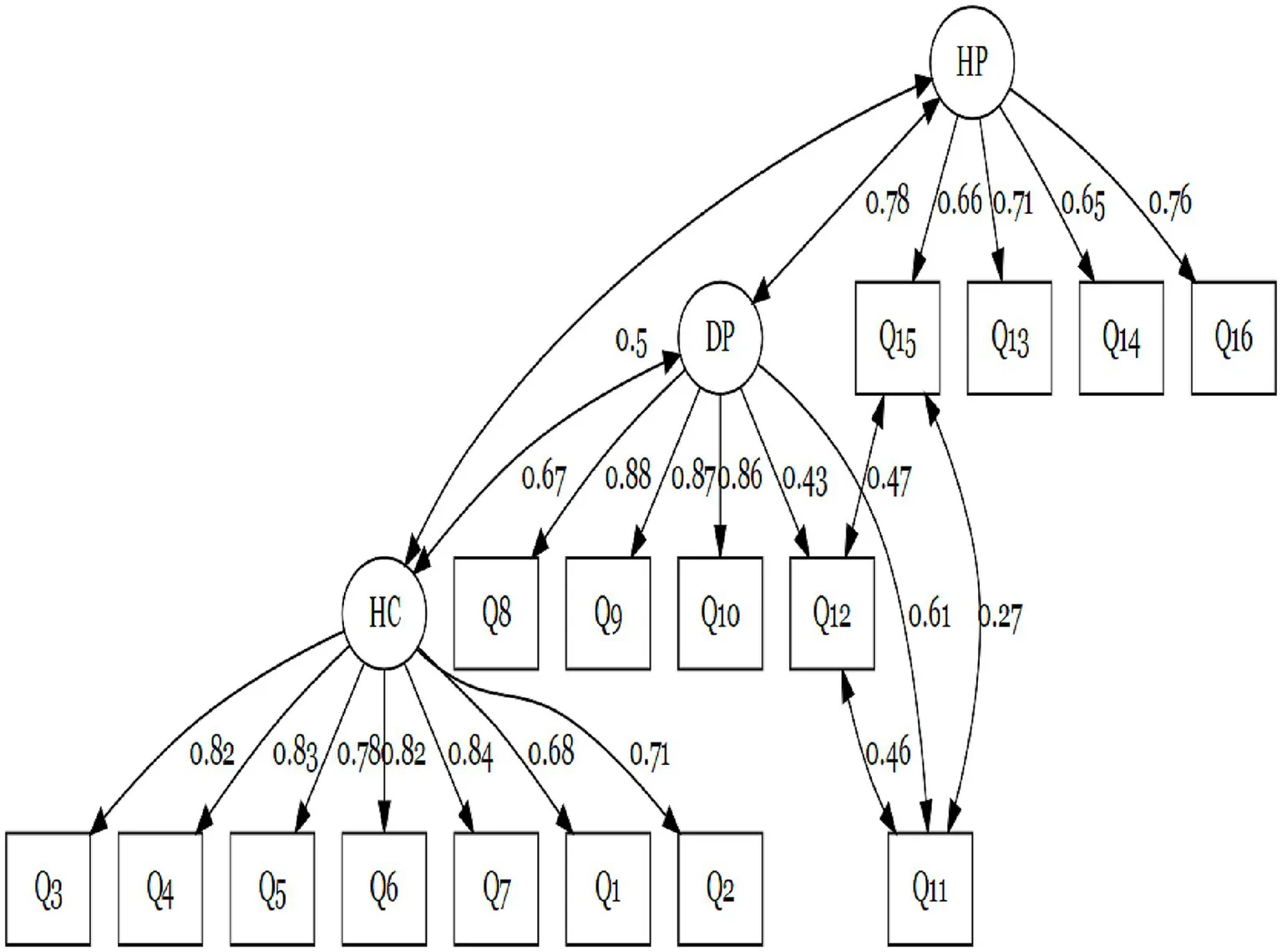
Structural equation model of health literacy factors (model 2.2). HC, Health care; DP, Disease prevention; HP, Health promotion.

The unidimensional polytomous item models showed only poor fit, while the three-dimensional models showed slightly better, yet overall still inadequate fit (Table 4). Standardized factor loadings of manifest variables on all three latent variables were moderate to high (Model 2.1: HC: 0.67–0.84, DP: 0.67–0.86, HP: 0.63–0.78; Model 2.2: HC: 0.68–0.84, DP: 0.43–0.88, HP: 0.65–0.76, Supplementary Tables 8,9). The correlations between the three latent factors (HC, DP, and HP) ranged from 0.50–0.84 and 0.50–0.78 for Model 2.1 and Model 2.2, respectively, suggesting a common dimension of HL (Figure 1).

Overall, the three-factor models demonstrated a substantially better fit than the unidimensional models, suggesting that a three-factor structure of HL (HC, HP, DP) provides a more robust representation of the data. However, in all models, the SRMR values remained well above the recommended cut-off, indicating persistent limitations in model fit. This is in contrast with previous research on the HLS-EU-Q and prompted further exploratory model evaluation in the present study. For this purpose, we fitted a bi-factor model following suggestions from previous studies investigating the structure of HL instruments (Elsworth et al., 2022; Wirtz et al., 2022). Bi-factor models allow simultaneous assessment of a general factor and domain-specific factors, with each item loading on the general factor and on one specific factor (Gibbons and Hedeker, 1992; Holzinger and Swineford, 1937). We tested a bi-factor model with correlated specific HL factors (HC, DP, HP) including correlated residuals between the three media-related items (i.e., Model 3). The introduced bi-factor model resulted in excellent fit and representation of the data (Table 4; Supplementary Table 10).

## 4 Discussion

This study aimed to assess HL among older patients in a clinical setting after elective surgery and to evaluate the psychometric properties of the HLS-EU-Q16 in this context. To our knowledge, this is the first study in Germany specifically focusing on HL in elderly patients after elective surgery within a clinical setting. Our findings align with previous research indicating limited HL in the elderly (Vogt et al., 2018; Vogt et al., 2020; Akubire et al., 2025; Słońska et al., 2015; Bozkurt and Demirci, 2019; Schaeffer et al., 2021a,b). However, contrary to prior population-based studies, we were unable to fully replicate the proposed three-factor model of HL (i.e., domains of HC, DP, and HP) due to poor model fit (Sørensen et al., 2013; Finbråten et al., 2018; Konopik et al., 2021).

### 4.1 Associations with health literacy

We found no statistically significant differences in overall HL, its domains, or HL skills (finding, understanding, appraising, and applying health information) across age groups. These findings contrast with those reported in one of the few German studies in this field involving data from a large German population-based study (HLS-GER), which revealed significantly lower HL among adults over 76 years compared to those under 76 (Vogt et al., 2020; Schaeffer et al., 2016). Several factors may explain this discrepancy: First, random sampling variation given our small sample size likely affected our results limiting generalizability. Second, our participants represent a highly selective group of older adults undergoing elective surgery. Older patients eligible for surgery may show higher levels of cognitive and physical fitness than their peers in the general population as they have been medically cleared for surgery. As frailty is associated with higher risk of mortality and poorer post-operative outcomes (Lin et al., 2016; Oakland et al., 2016), existing high frailty may have been a reason to postpone or cancel elective surgery. The frailest individuals may therefore be underrepresented, which could have led to a more uniform sample and may partially explain the absence of group differences in HL across age subgroups, thus also limiting variability in our HL measure. Third, compared with the heterogeneous, population-based HLS-GER sample, our participants were assessed in a structured hospital context with preoperative information and repeated interactions with healthcare professionals. This environment may have promoted engagement with health information and contributed to higher observed HL levels (Brodersen et al., 2023). Fourth, central methodological differences also limit comparability. We used the short form of the HLS-EU-Q, which captures fewer dimensions than the full HLS-EU-Q47. Additionally, differences in age group categorization and the relatively small sample size may have reduced the sensitivity of subgroup analyses.

Domain-specific comparisons with HLS-GER data revealed further differences (Vogt et al., 2020; Schaeffer et al., 2016). We found no statistically significant differences between age groups across the three different HL domains or the four skill levels of HL. However, Vogt et al. reported statistically significant lower mean values in all three domains as well as all four skill levels of HL among over 75-year old compared to younger individuals (Vogt et al., 2020). These comparisons, however, should be interpreted cautiously due to differences in sampling methods and sample sizes, age group definitions, and the fundamental contrast between hospital-based and community-based settings. Overall, these findings highlight the context-specific nature of HL and underscore the potential limits in comparing community-based and clinical populations, emphasizing the need for further research on HL among older adults across various environments. Additionally, we observed no statistically significant differences in mean HL scores across gender, education, or monthly net household income, consistent with the mixed findings for this population that were reported in prior research (Tiller et al., 2015; Vogt et al., 2018; Eronen et al., 2018; Lorini et al., 2019). However, lower HL levels in older patients were associated with higher symptoms of anxiety and depression, aligning with existing evidence linking limited HL to increased psychological distress and suggesting that improving HL could mitigate depression and anxiety symptoms (Haeri-Mehrizi et al., 2024; Magallón-Botaya et al., 2023; Demirel et al., 2023).

### 4.2 Psychometric evaluation

Our CFA results showed difficulties in replicating the three-factor structure of the HLS-EU-Q16 in this clinical sample, primarily reflected in poor model fit. However, factor loadings remained high, indicating the questionnaire measures a common underlying HL construct. Notably, SRMR and RMSEA values in all our unidimensional and three-dimensional models remained well above the cut-offs. While high SRMR and RMSEA values may partly be attributable to the small sample size, elevated values across all models suggest model misspecification (Kenny et al., 2015), potentially attributable to still unexplained residual covariance. Notably, overall model fit improved in specified models by including correlated residuals of media-related items, which suggests that these items share unexplained variance not reflected by the original three-factor model of the HLS-EU-Q16, and may indicate a distinct media factor, also described in other studies (Gustafsdottir et al., 2020; Lamot and Kirbiš, 2024; Coman et al., 2022). Interestingly, our exploratorily fitted bi-factor model including both a general factor of HL and the three distinct HL domain factors (HC, DP, HP), while also adding correlated residuals of the three media-related items, showed excellent fit according to all global fit indices. While this should be interpreted very cautiously due to the pure exploratory nature of the step, it may offer a useful analytic framework for future studies examining HL in specific groups and environments. Another explanation for the overall poor model fit of our primary models could be that contextual factors may indeed have contributed to the observed differences in factor structure in this study. Patients in this study were assessed in a perioperative hospital setting, characterized by, for example, increased stress as well as repeated contact with HCPs. These contextual conditions may have shaped how patients answered the questionnaire and how HL was expressed, potentially affecting the underlying factor structure (Goretzko et al., 2023). These findings contribute to the existing body of literature, which also reports challenges in reproducing the original HLS factor structure across different contexts (e.g., different cultures) and populations (e.g., older adults). In particular, studies on the 16-item short form have shown mixed results (Gustafsdottir et al., 2020; Lamot and Kirbiš, 2024; Bergman et al., 2023; Finbråten et al., 2018; Coman et al., 2022; Tiller et al., 2015; Bas-Sarmiento et al., 2020).

Our analysis also revealed substantial difficulties and high proportions of “do not know” responses among older patients in responding to media-related HLS-EU-Q16 items (Q11, Q12, Q15). Different explanations should be considered. First, challenges in navigating media-based information among older adults are consistent with earlier research documenting limited digital HL in this population (Lorini et al., 2019; Meier et al., 2022; Gustafsdottir et al., 2022; Hoffman, 2026). This is particularly striking for Germany in 2026, where recent data still show that 10% of individuals aged 65–74 have never used the internet (Statistisches Bundesamt, 2026). In addition, a recent preprint reported high rates of low digital HL among Swiss adults over 75 (Meier et al., 2022), and decade long HL studies from Germany have similarly shown low digital HL, although with slight improvements in recent years (Schaeffer et al., 2025; Hurrelmann et al., 2022; Deutscher Ärzteverlag GmbH, 2025; Schaeffer et al., 2021b). These findings are particularly relevant in light of the COVID-19 pandemic, which amplified both the volume and complexity of health-related information (the so-called infodemic). Second, contextual factors of the perioperative setting may have also impacted response patterns on these specific items. In perioperative situations, surgical patients often experience heightened anxiety and stress (Demirel et al., 2023; Kassahun et al., 2022; Abate et al., 2020), as also indicated by our findings. This situation can lead to cognitive overload, which may also impair information processing (Shebl et al., 2025; Oteri et al., 2021). Consequently, older patients may rely primarily on easier, more accessible information sources, such as direct communication with HCPs rather than independent digital information seeking (Keenan et al., 2024), potentially pointing to a reduced relevance of media-related items. In combination with the limited digital (health) literacy, ensuring adequate non-digital alternatives and plain language communication during the perioperative period can be crucial to avoid further burdening this patient group (Choolayil et al., 2024).

### 4.3 Implications

Overall, our findings contribute to the research field of HL in Germany and to more recent developments in HL research, which expand concepts of individual HL to the more systemic approach of organizational HL (OHL). Especially in healthcare settings, this research focuses on reducing system barriers while establishing HL at an organizational level, while also focusing on the role of HCPs through professional HL (Sampson and Sampson, 2026; Bremer et al., 2021). Considering its context-dependency, HL is not limited to patient capabilities, and should not be reduced to individual responsibility but be reflected in light of situational demands, such as the perioperative setting in our study (Sampson and Sampson, 2026). Low HL can be an additional barrier in already vulnerable groups as it often affects populations with already higher risk for adverse clinical outcomes [e.g., postoperative complications (Theiss et al., 2022)] and higher clinical needs [e.g., increased nursing complexity (Cocchieri et al., 2025)]. Routine clinical HL screening, for example integrated in pre-operative geriatric risk assessments (Ron et al., 2026; Oresanya et al., 2014), may help address individual patient needs early on and enable more tailored, individualized care approaches that could ultimately improve overall clinical outcomes more equitably. Importantly, before implementing such screening, further investigation and adjustments of current HL instruments may be warranted. As our findings indicate, different contexts may impact response patterns differently, potentially leading to over- or underestimating individual HL, which may complicate finding adequate individual support measures, particularly in the context of intersectoral vulnerability (e.g., after hospital discharge). This was also previously discussed with regard to the subjective nature of the self-reported HLS-EU-Qs (Finbråten et al., 2018; Wirtz et al., 2022). When answering questions about HL, specific patient groups, compared to the general population, may rely on other reference frames based on their experiences with managing their health and using healthcare structures. Accordingly, experience and context may jointly impact response patterns. Overall, HL screenings should be seen as a basis for further dialogue with the patient, rather than a clinical decision maker. Raising awareness of possible HL implication among HCPs in clinical settings may further strengthen patient-centered care approaches (Bavin et al., 2023). This seems particularly important as promoting professional HL among HCPs is a very new approach in Germany, and first studies show limited familiarity with the HL concept among HCPs, even though their professional HL is moderately high and supporting techniques, such as Teach-Back, are often known (Griese and Schaeffer, 2025). Accordingly, the development and implementation of more comprehensive HL approaches that take into account the patient situation as well as the competencies of HCPs and systemic aspects of healthcare structures, can ultimately benefit everyone. Furthermore, future studies should replicate our findings in larger clinical populations, including longitudinal study designs. Examining potential measurement discrepancies resulting from other contexts surrounding elective surgery, such as post-discharge situations to patient homes or care facilities, could also be promising research approaches.

### 4.4 Strength and limitation

Our study focused specifically on HL levels among older adults after elective surgery, contributing to the limited evidence on HL within clinical settings for this demographic group. While most HL research is population-based, healthcare-specific contexts remain underrepresented. Our findings highlight the complexities and challenges associated with assessing HL in specialized clinical environments. Furthermore, the use of a self-report measure allowed direct insight into patients´ perceived abilities in handling health information. A key strength of this study is the rigorous validation approach using confirmatory factor analysis (CFA).

Our study also has several limitations. The relatively small sample size limits the precision of estimates and renders group comparisons and correlation analyses underpowered for detecting small to moderate effects. This limitation is particularly notable in the context of our CFA, where the relatively small sample size may have reduced the stability of factor estimates and limited the precision of the overall model. Accordingly, the presented findings should be interpreted with caution, and replication with larger samples in similar clinical contexts are necessary to underscore these results and further clarify if the HLS-EU-Q16 requires clinical adjustments and whether alternative factor structures better reflect HL among older hospital patients. Furthermore, the cross-sectional design limit the interpretability of the findings and preclude causal conclusions. Unanticipated interruptions in data collection due to the COVID-19 pandemic may have influenced patient recruitment and responses, although no significant differences in HL levels and most sociodemographic variables were detected before and after the lockdowns. However, the statistically significant differences in age between both recruitment groups limit overall study interpretation. While no systematic age bias in HL was found in the age-stratified sensitivity analyses, pointing to the appropriateness of pooling both samples, we acknowledge that recruitment context itself (pre- vs. post-pandemic lockdowns) may have introduced unknown bias. Although identical inclusion and exclusion criteria were applied for both samples, the pandemic may have led to differences in the patient population. Additionally, selection bias resulting from the specific inpatient population undergoing elective surgery is possible. Finally, residual confounding cannot be fully excluded.

Despite these limitations, our primary aim was to examine HL among older patients in the specific setting of elective surgery and to evaluate the psychometric properties of the HLS-EU-Q16 in a real-life clinical environment. Given the described limitations and knowledge gaps surrounding HL in vulnerable surgical populations, we believe our work can inspire future research in this area.

## 5 Conclusion

HL is a dynamic construct that varies over time and is strongly shaped by contextual factors. Our findings highlight both the importance and potential distinct characteristics of addressing HL in older adults, particularly during high-risk periods such as the perioperative phase. Our inability to fully replicate the hypothesized HLS-EU-Q16 structure adds to growing evidence of variability across specific participant groups and study contexts in this area. Future research should further evaluate dimensionality of existing HLS-EU-Qs in larger samples to capture the potentially unique demands and information needs of particular healthcare scenarios. Building on this, HL interventions in this field should be more explicitly tailored to the specific demands and capabilities of the target population and context and should include more comprehensive HL approaches including patients, HCPs, and the surrounding systems. Additionally, our study underscores the need for greater methodological standardization, including clearer definitions of age subgroups, to enhance the comparability of HL research across studies.

## Data Availability

The datasets presented in this article are not readily available because all data relevant to the study are included in the article or uploaded as supplemental information. Requests to access the datasets should be directed to mo.schoenfeld@posteo.de.
