# Supplementary material for: Measuring health literacy among older inpatients after elective surgery: evaluation of the European Health Literacy Survey Questionnaire-short form (HLS-EU-Q16)
Source: Front Psychol. 2026 Aug 28;17:1935428. doi: 10.3389/fpsyg.2026.1935428 (PMC13561981; doi:10.3389/fpsyg.2026.1935428)
Supplement: Supplementary file 1 [file Table_1.DOCX]

**Supplementary Table 1**. Levels of Health Literacy Across the Three Dimensions of the HLS-EU-Q16 in 143 Elderly Inpatients After Elective Surgery.

| **Age Group** |  | **60-69 years-old** | | **70-79 years-old** | | **≥80 years-old** | |  | **Total sample** | |
| --- | --- | --- | --- | --- | --- | --- | --- | --- | --- | --- |
|  | | | | | | | | |  |  |
|  |  | **Mean (SD)** | **[CI]** | **Mean (SD)** | **[CI]** | **Mean (SD)** | **[CI]** | **p** | **Mean (SD)** | **[CI]** |
| **Health Care** |  | 31.59 (9.75) | 28.69-34.49 | 34.75 (8.42) | 32.80-36.70 | 34.02 (7.94) | 30.20-37.85 | .391^†^ | 33.67 (8.88) | 32.19-35.15 |
|  |  | **n** | **%** | **n** | **%** | **n** | **%** |  | **n** | **%** |
| Limited | Inadequate | 9 | 19.6 | 9 | 12.2 | 3 | 15.8 |  | 21 | 15.1 |
|  | Problematic | 11 | 23.9 | 20 | 27.0 | 1 | 5.3 |  | 32 | 23.0 |
| Adequate | Sufficient | 22 | 47.8 | 28 | 37.8 | 12 | 63.2 |  | 62 | 44.6 |
|  | Excellent | 4 | 8.7 | 17 | 23.0 | 3 | 15.8 |  | 24 | 17.3 |
|  | | | | | | | | |  |  |
|  |  | **Mean (SD)** | **[CI]** | **Mean (SD)** | **[CI]** | **Mean (SD)** | **[CI]** | **p** | **Mean (SD)** | **[CI]** |
| **Disease Prevention** |  | 32.97 (8.61) | 30.36-35.59 | 32.84 (8.07) | 30.88-34.82 | 30.06 (8.65) | 24.84-35.29 | .580^†^ | 32.66 (8.30) | 31.19-34.13 |
|  |  | **n** | **%** | **n** | **%** | **n** | **%** |  | **n** | **%** |
| Limited | Inadequate | 7 | 15.9 | 13 | 19.4 | 3 | 23.1 |  | 23 | 18.5 |
|  | Problematic | 10 | 22.7 | 17 | 25.4 | 3 | 23.1 |  | 30 | 24.2 |
| Adequate | Sufficient | 21 | 47.7 | 28 | 41.8 | 6 | 46.2 |  | 55 | 44.4 |
|  | Excellent | 6 | 13.6 | 9 | 13.4 | 1 | 7.7 |  | 16 | 12.9 |
|  | | | | | | | | |  |  |
|  |  | **Mean (SD)** | **[KI]** | **Mean (SD)** | **[CI]** | **Mean (SD)** | **[CI]** | **p** | **Mean (SD)** | **[CI]** |
| **Health Promotion** |  | 33.11 (8.88) | 30.38-38.84 | 33.21 (9.53) | 30.96-35.47 | 31.84 (5.21) | 28.97-34.74 | .814^†^ | 33.03 (8.94) | 31.48-34.57 |
|  |  | **n** | **%** | **n** | **%** | **n** | **%** |  | **n** | **%** |
| Limited | Inadequate | 8 | 18.6 | 18 | 25.4 | 2 | 13.3 |  | 28 | 21.7 |
|  | Problematic | 9 | 20.9 | 10 | 14.1 | 4 | 26.7 |  | 23 | 17.8 |
| Adequate | Sufficient | 20 | 46.5 | 31 | 43.7 | 9 | 60.0 |  | 60 | 46.5 |
|  | Excellent | 6 | 14.0 | 12 | 16.9 | 0 | 0.0 |  | 18 | 14.0 |

**Notes**. ^†^Kruskal-Wallis H Test. Sample size varies due to missing values.

**Supplementary Table 2**. Distribution of Health Literacy Levels According to the Four Skill Categories in 143 Elderly Inpatients After Elective Surgery.

| **Age Group** |  | **60-69 years-old** | | **70-79 years-old** | | **≥80 years-old** | |  | **Total Sample** | |
| --- | --- | --- | --- | --- | --- | --- | --- | --- | --- | --- |
|  | | | | | | | | |  |  |
|  |  | **Mean (SD)** | **[CI]** | **Mean (SD)** | **[CI]** | **Mean (SD)** | **[CI]** | **p** | **Mean (SD)** | **[CI]** |
| **Find** |  | 29.83 (9.88) | 26.75-32.91 | 33.17 (8.87) | 31.04-36.00 | 30.65 (7.07) | 26.57-34.74 | .314^†^ | 31.78 (9.07) | 30.18-33.38 |
|  |  | **n** | **%** | **n** | **%** | **n** | **%** |  | **n** | **%** |
| Limited | Inadequate | 15 | 35.7 | 15 | 21.7 | 3 | 21.4 |  | 33 | 26.4 |
|  | Problematic | 7 | 16.7 | 13 | 18.8 | 3 | 21.4 |  | 23 | 18.4 |
| Adequate | Sufficient | 17 | 40.5 | 31 | 44.9 | 7 | 50.0 |  | 55 | 44.0 |
|  | Excellent | 3 | 7.1 | 10 | 14.5 | 1 | 7.1 |  | 14 | 11.2 |
|  | | | | | | | | |  |  |
|  |  | **Mean (SD)** | **[CI]** | **Mean (SD)** | **[CI]** | **Mean (SD)** | **[CI]** | **p** | **Mean (SD)** | **[CI]** |
| **Understand** |  | 36.18 (7.44) | 33.89-38.46 | 37.20 (7.45) | 35.45-38.95 | 34.55 (8.59) | 29.97-39.12 | .739^†^ | 36.62 (7.61) | 35.31-37.93 |
|  |  | **n** | **%** | **n** | **%** | **n** | **%** |  | **n** | **%** |
| Limited | Inadequate | 3 | 7.0 | 4 | 5.6 | 2 | 12.5 |  | 9 | 6.9 |
|  | Problematic | 6 | 14.0 | 16 | 22.2 | 1 | 6.3 |  | 23 | 17.6 |
| Adequate | Sufficient | 27 | 62.8 | 32 | 44.4 | 11 | 68.8 |  | 70 | 53.4 |
|  | Excellent | 7 | 16.3 | 20 | 27.8 | 2 | 12.5 |  | 29 | 22.1 |
|  | | | | | | | | |  |  |
|  |  | **Mean (SD)** | **[CI]** | **Mean (SD)** | **[CI]** | **Mean (SD)** | **[CI]** | **p** | **Mean (SD)** | **[CI]** |
| **Appraise** |  | 29.23 (9.82) | 26.17-32.29 | 30.24 (8.21) | 28.18-32.31 | 27.78 (8.61) | 23.19-32.36 | .646^†^ | 29.70 (8.89) | 28.10-31.28 |
|  |  | **n** | **%** | **n** | **%** | **n** | **%** |  | **n** | **%** |
| Limited | Inadequate | 14 | 33.3 | 15 | 23.8 | 5 | 31.3 |  | 34 | 28.1 |
|  | Problematic | 7 | 16.7 | 18 | 28.6 | 5 | 31.3 |  | 30 | 24.8 |
| Adequate | Sufficient | 17 | 40.5 | 26 | 41.3 | 5 | 31.3 |  | 48 | 39.7 |
|  | Excellent | 4 | 9.5 | 4 | 6.3 | 1 | 6.3 |  | 9 | 7.4 |
|  | | | | | | | | |  |  |
|  |  | **Mean (SD)** | **[CI]** | **Mean (SD)** | **[CI]** | **Mean (SD)** | **[CI]** | **p** | **Mean (SD)** | **[CI]** |
| **Apply** |  | 30.68 (9.24) | 27.87-33.49 | 31.94 (9.02) | 29.76-34.13 | 31.60 (9.23) | 26.68-36.52 | .908^†^ | 31.57 (9.11) | 29.98-33.15 |
|  |  | **n** | **%** | **n** | **%** | **n** | **%** |  | **n** | **%** |
| Limited | Inadequate | 9 | 20.5 | 14 | 20.6 | 3 | 18.8 |  | 26 | 20.3 |
|  | Problematic | 13 | 29.5 | 16 | 23.5 | 5 | 31.3 |  | 34 | 26.6 |
| Adequate | Sufficient | 17 | 38.6 | 29 | 42.6 | 6 | 37.5 |  | 52 | 40.6 |
|  | Excellent | 5 | 11.4 | 9 | 13.2 | 2 | 12.5 |  | 16 | 12.5 |

**Notes**. ^†^Kruskal-Wallis H Test. Sample size varies due to missing values.

**Supplementary table 3.** Spearman Correlations Between HLS-EU-Q16 Items in 143 Elderly Inpatients After Elective Surgery.

|  | **Q1** | **Q2** | **Q3** | **Q4** | **Q5** | **Q6** | **Q7** | **Q8** | **Q9** | **Q10** | **Q11** | **Q12** | **Q13** | **Q14** | **Q15** | **Q16** |
| --- | --- | --- | --- | --- | --- | --- | --- | --- | --- | --- | --- | --- | --- | --- | --- | --- |
| **Q1** | 1 |  |  |  |  |  |  |  |  |  |  |  |  |  |  |  |
| **Q2** | .475** | 1 |  |  |  |  |  |  |  |  |  |  |  |  |  |  |
| **Q3** | .352** | .486** | 1 |  |  |  |  |  |  |  |  |  |  |  |  |  |
| **Q4** | .308** | .420** | .551** | 1 |  |  |  |  |  |  |  |  |  |  |  |  |
| **Q5** | .408** | .368** | .553** | .363** | 1 |  |  |  |  |  |  |  |  |  |  |  |
| **Q6** | .523** | .442** | .525** | .465** | .573** | 1 |  |  |  |  |  |  |  |  |  |  |
| **Q7** | .336** | .462** | .552** | .577** | .445** | .511** | 1 |  |  |  |  |  |  |  |  |  |
| **Q8** | .491** | .370** | .438** | .394** | .564** | .522** | .381** | 1 |  |  |  |  |  |  |  |  |
| **Q9** | .174 | .184* | .123 | .406** | .103 | .206* | .402** | .349** | 1 |  |  |  |  |  |  |  |
| **Q10** | .131 | .257** | .228** | .380** | .137 | .183* | .310** | .175 | .640** | 1 |  |  |  |  |  |  |
| **Q11** | .229* | .165 | .310** | .358** | .406** | .389** | .363** | .451** | .270** | .154 | 1 |  |  |  |  |  |
| **Q12** | .114 | .125 | .206* | .212* | .194* | .218* | .173* | .232* | .290** | .172* | .648** | 1 |  |  |  |  |
| **Q13** | .333** | .310** | .243** | .308** | .191* | .270** | .239** | .435** | .380** | .345** | .253** | .292** | 1 |  |  |  |
| **Q14** | .191* | .174 | .099 | .210* | .206* | .222* | .152 | .331** | .390** | .289** | .301** | .348** | .282** | 1 |  |  |
| **Q15** | .153 | .188* | .169 | .196* | .245** | .269** | .309** | .295** | .309** | .215* | .492** | .586** | .302** | .502** | 1 |  |
| **Q16** | .277** | .226** | .178* | .238** | .201* | .243** | .222** | .251* | .432** | .457** | .210* | .241** | .439** | .448** | .377** | 1 |

**Notes**. *p<0.05; **p<0.01; Details on item content and domains are provided in Table 2 of the main text.

**Supplementary table 4.** Spearman Correlations Between HL Scores

and Dimensions in 143 Elderly Inpatients After Elective Surgery.

|  | **G-HLS** | **G-HLS: HC** | **G-HLS: DP** | **G-HLS: HP** |
| --- | --- | --- | --- | --- |
| **G-HLS** | 1 |  |  |  |
| **G-HLS: HC** | .855^**^ | 1 |  |  |
| **G-HLS: DP** | .838^**^ | .551^**^ | 1 |  |
| **G-HLS: HP** | .712^**^ | .387^**^ | .622^**^ | 1 |

**Notes**. *p<0.05; **p<0.01. **G-HLS**: HL index score; **HC**: Health Care;

**DP**: Disease Prevention; **HP**: Health Promotion.

**Supplementary table 5.** Associations Between Health Literacy and Sociodemographic as well as Health Outcomes in 143 Elderly Inpatients After Elective Surgery.

|  | **n** | **Mean (SD)** | **[CI]** | **Median (Q1, Q3)** | ***p* value** |
| --- | --- | --- | --- | --- | --- |
| **Gender** |  |  |  |  | .97^†^ |
| Male | 67 | 32.8 (6.4) | 31.3-34.4 | 33.3 (30.2, 31.2) |  |
| Female | 60 | 33.3 (7.9) | 31.3-35.3 | 31.8 (28.1, 40.6) |  |
| **Age group** |  |  |  |  | .70^††^ |
| 65-69 | 44 | 32.4 (7.7) | 30.1-34.8 | 32.8 (29.2, 38.3) |  |
| 70-79 | 70 | 33.9 (7.0) | 32.2-35.5 | 33.3 (28.9, 39.7) |  |
| ≥80 | 16 | 31.9 (6.5) | 28.5-35.4 | 32.3 (28.6, 35.5) |  |
| **Education level** |  |  |  |  | .38^††^ |
| Low | 36 | 32.8 (8.6) | 29.9-35.7 | 32.8 (26.2, 40.0) |  |
| Medium | 63 | 33.9 (5.9) | 32.4-35.4 | 33.3 (30.8, 38.9) |  |
| High | 30 | 31.6 (7.6) | 28.7-34.4 | 31.8 (26.4, 36.0) |  |
| **Income** |  |  |  |  | .96^†^ |
| Below average (<4.000 EUR) | 66 | 32.9 (7.2) | 31.4-34.5 | 33.3 (27.9, 39.2) |  |
| Above average (≥4.000 EUR) | 46 | 32.3 (7.4) | 29.2-35.4 | 33.3 (28.1, 37.8) |  |
| **PHQ-4** |  |  |  |  | **.02*^††^** |
| No distress | 63 | 34.8 (7.4) | 33.0-36.7 | 35.4 (31.0, 40.5) |  |
| Mild distress | 37 | 33.4 (6.3) | 31.3-35.5 | 33.3 (29.2, 38.0) |  |
| Moderate distress | 15 | 29.7 (4.8) | 27.0-32.3 | 30.2 (27.1, 34.4) |  |
| Severe distress | 7 | 28.7 (10.7) | 18.8-38.6 | 28.1 (15.6, 34.4) |  |
| **MARS-D** |  |  |  |  | .62^†^ |
| Non-adherent | 26 | 32.0 (6.4) | 29.4-34.6 | 32.8 (27.9, 36.5) |  |
| Adherent | 102 | 33.3 (7.3) | 31.8-34.7 | 33.3 (29.2, 36.5) |  |

**Notes**. *p<.05, ^†^Mann-Whitney U Test; ^††^Kruskal-Wallis H Test; **G-HLS**: HL index score;

**HLS scores**: Higher scores indicate higher HL. Sample sizes differ due to missing values.

**Supplementary table 6.** Spearman Correlations Among Outcomes in a Sample of 143 Elderly Inpatients After Elective Surgery.

|  | **G-HLS-Q16** | **G-HLS-Q16 HC** | **G-HLS-Q16 DP** | **G-HLS-Q16 HP** | **Medication Adherence** | **Number of drugs** | **Depression** | **Anxiety** | **Physical Health** | **Mental Health** | **Cognitive Function** |
| --- | --- | --- | --- | --- | --- | --- | --- | --- | --- | --- | --- |
| **G-HLS-Q16**  (HLS-EU-Q16) | 1 |  |  |  |  |  |  |  |  |  |  |
| **G-HLS-Q16 HC**  (HLS-EU-Q16) | .855** | 1 |  |  |  |  |  |  |  |  |  |
| **G-HLS-Q16 DP**  (HLS-EU-Q16) | .838** | .551** | 1 |  |  |  |  |  |  |  |  |
| **G-HLS-Q16 HP**  (HLS-EU-Q16) | .712** | .387** | .622** | 1 |  |  |  |  |  |  |  |
| **Med. Adherence** (MARS-D) | .142 | .194* | .138 | -.003 | 1 |  |  |  |  |  |  |
| **Number of**  **drugs** | -.131 | -.109 | -.144 | -.026 | .045 | 1 |  |  |  |  |  |
| **Depression**  (PHQ-4) | -.285** | -.211* | -.190* | -.287** | -.131 | .112 | 1 |  |  |  |  |
| **Anxiety**  (PHQ-4) | -.310** | -.310** | -.194* | -.226* | -.166 | .037 | .766** | 1 |  |  |  |
| **Physical Health**  (SF-12) | .277** | .232* | .216* | .253* | .179 | -.203 | -.376** | -.340** | 1 |  |  |
| **Mental Health**  (SF-12) | .325** | .277** | .214* | .165 | .228* | -.035 | -.656** | -.630** | .188 | 1 |  |
| **Cognitive Function** (WHODAS 2.0) | -.333** | -.283** | -.250** | -.238** | -.122 | .113 | .591** | .482** | -.463** | -.423** | 1 |

Notes. *p<0.05; **p<0.01; **HC**: Health Care; **DP**: Disease Prevention; **HP**: Health Promotion.

**Supplementary table 7.** Descriptive Statistics of Health-Related Outcomes

in a Sample of 143 Elderly Inpatients After Elective Surgery.

| **Item** | **Mean** | **SD** | **Min-Max** |  |
| --- | --- | --- | --- | --- |
| PHQ-4 total score | 2.9 | 2.8 | 0-12 |  |
| Depression (PHQ-4) | 1.6 | 1.5 | 0-6 |  |
| Anxiety (PHQ-4) | 1.4 | 1.5 | 0-6 |  |
| Medication adherence (MARS-D) | 23.5 | 1.8 | 15-25 |  |
| Number of drugs taken regularly | 5.5 | 3.6 | 0-17 |  |
| Physical health (PCS, SF-12) | 35.9 | 10.5 | 16.3-56.8 |  |
| Mental health (MCS, SF-12) | 48.8 | 11.9 | 22.8-67.5 |  |
| WHODAS 2.0 | 9.9 | 4.0 | 6-24 |  |

**Note**s. *p<0.05; **p<0.01

**MARS-D**: higher scores indicate higher medication adherence; **PHQ**: higher scores indicate higher levels of depression and anxiety;

**SF-12**: higher scores indicate better physical and mental health; **WHODAS**: higher scores indicate higher cognitive disability.

**Supplementary table 8.** Factor Loadings of the Unidimensional CFA Models in a Sample

of 143 Elderly Patients After Elective Surgery.

| **Item** | **Factor** | **Model** | **Std. Loading** | **Residual Variance** | **Model** | **Std. Loading** | **Residual Variance** |
| --- | --- | --- | --- | --- | --- | --- | --- |
| **Q1** | HL | Model 1.1 | .623 | .612 | Model 1.2^†^ | .638 | .593 |
| **Q2** | HL |  | .640 | .591 |  | .656 | .570 |
| **Q3** | HL |  | .762 | .420 |  | .774 | .400 |
| **Q4** | HL |  | .746 | .444 |  | .757 | .427 |
| **Q5** | HL |  | .726 | .473 |  | .741 | .451 |
| **Q6** | HL |  | .761 | .422 |  | .776 | .397 |
| **Q7** | HL |  | .781 | .390 |  | .796 | .367 |
| **Q8** | HL |  | .747 | .443 |  | .761 | .421 |
| **Q9** | HL |  | .780 | .392 |  | .788 | .378 |
| **Q10** | HL |  | .781 | .389 |  | .790 | .376 |
| **Q11** | HL |  | .695 | .516 |  | .547 | .701 |
| **Q12** | HL |  | .573 | .672 |  | .361 | .870 |
| **Q13** | HL |  | .545 | .703 |  | .554 | .694 |
| **Q14** | HL |  | .485 | .765 |  | .481 | .768 |
| **Q15** | HL |  | .608 | .630 |  | .496 | .754 |
| **Q16** | HL |  | .572 | .673 |  | .575 | .669 |

**Notes**: ^†^Correlated residuals between Q11-Q12, Q11-Q15, Q12-Q15 (i.e. media-related items) were included; **HL**: Health Literacy. Details on item content and domains are provided in Table 2 of the main text.

**Supplementary table 9.** Factor Loadings of the Three-Dimensional CFA Models in a Sample

of 143 Elderly Patients After Elective Surgery.

| **Item** | **Factor** | **Model** | **Std. Loading** | **Residual Variance** | **Model** | **Std. Loading** | **Residual Variance** |
| --- | --- | --- | --- | --- | --- | --- | --- |
| **Q1** | HC | Model 2.1 | .673 | .547 | Model 2.2^†^ | .675 | .544 |
| **Q2** | HC |  | .704 | .505 |  | .705 | .503 |
| **Q3** | HC |  | .815 | .336 |  | .815 | .336 |
| **Q4** | HC |  | .829 | .313 |  | .825 | .319 |
| **Q5** | HC |  | .774 | .400 |  | .775 | .399 |
| **Q6** | HC |  | .821 | .326 |  | .821 | .326 |
| **Q7** | HC |  | .838 | .297 |  | .838 | .297 |
| **Q8** | DP |  | .842 | .290 |  | .884 | .219 |
| **Q9** | DP |  | .858 | .264 |  | .873 | .238 |
| **Q10** | DP |  | .849 | .279 |  | .859 | .262 |
| **Q11** | DP |  | .771 | .405 |  | .615 | .622 |
| **Q12** | DP |  | .666 | .556 |  | .431 | .814 |
| **Q13** | HP |  | .663 | .561 |  | .711 | .494 |
| **Q14** | HP |  | .628 | .606 |  | .651 | .577 |
| **Q15** | HP |  | .780 | .392 |  | .665 | .558 |
| **Q16** | HP |  | .719 | .483 |  | .757 | .427 |

**Notes**: ^†^Correlated residuals between Q11-Q12, Q11-Q15, Q12-Q15 (i.e. media-related items) were included. **HC**: Health Care, **DP**: Disease Prevention, **HP**: Health Promotion. Details on item content and domains are provided in Table 2 of the main text.

**Supplementary table 10.** Factor Loadings of the correlated Bi-factor CFA Model in a Sample of 143 Elderly Patients After Elective Surgery.

| **Item** | **Factor** | **Std. Loading** | **Factor** | **Std. Loading** | **Residual Variance** |  |
| --- | --- | --- | --- | --- | --- | --- |
| **Q1** | HL | .653 | HC | .278 | .497 |  |
| **Q2** | HL | .514 | HC | .486 | .500 |  |
| **Q3** | HL | .629 | HC | .523 | .331 |  |
| **Q4** | HL | .422 | HC | .786 | .204 |  |
| **Q5** | HL | .805 | HC | .242 | .293 |  |
| **Q6** | HL | .722 | HC | .415 | .307 |  |
| **Q7** | HL | .488 | HC | .726 | .234 |  |
| **Q8** | HL | .738 | DP | .380 | .311 |  |
| **Q9** | HL | .005 | DP | .964 | .071 |  |
| **Q10** | HL | .002 | DP | .920 | .154 |  |
| **Q11** | HL | .507 | DP | .312 | .646 |  |
| **Q12** | HL | .195 | DP | .381 | .817 |  |
| **Q13** | HL | .377 | HP | .587 | .513 |  |
| **Q14** | HL | .252 | HP | .622 | .550 |  |
| **Q15** | HL | .349 | HP | .548 | .577 |  |
| **Q16** | HL | .237 | HP | .746 | .387 |  |

**Notes**: ^†^Correlated residuals between Q11-Q12, Q11-Q15, Q12-Q15 (i.e. media-related items) were included. **HC**: Health Care, **DP**: Disease Prevention, **HP**: Health Promotion. Details on item content and domains are provided in Table 2 of the main text.
